# Multiple Giant Liver Cysts in a Nepalese Lad

**DOI:** 10.1155/2020/6196252

**Published:** 2020-08-29

**Authors:** Ashish Lal Shrestha, Shova Banstola Paudel, Saurav Krishna Malla

**Affiliations:** ^1^Department of Pediatric Surgery, Grande International Hospital, Dhapasi, Kathmandu, Nepal; ^2^Department of Pathology, Grande International Hospital, Dhapasi, Kathmandu, Nepal; ^3^Department of Radio Diagnosis, Grande International Hospital, Dhapasi, Kathmandu, Nepal

## Abstract

**Background:**

While evaluating a child with multicystic liver pathology, both the congenital and acquired etiologies need to be considered. While typicality of findings on abdominal imaging makes the diagnosis of cystic echinococcosis relatively easy, choosing the appropriate line of management is equally crucial. *Case Presentation*. An 8-year-old previously healthy lad presented to the office with progressive upper abdominal fullness and pain for a year. Blood workup was normal. CT imaging made a diagnosis of multicystic giant hepatic hydatidosis. Treatment consisting of oral albendazole combined with surgical excision resulted in a fruitful outcome. To the best of our knowledge, this probably represents the first case of multiple giant hepatic hydatidosis at such a young age being reported from Nepal.

**Conclusion:**

Childhood liver cysts are uncommon. Considering the endemicity, echinococcal etiology needs consideration. Surgical ablation is required for large cysts, and the mode of management is dictated by the size and location.

## 1. Introduction

Echinococcal disease is an anthropozoonosis with sheep and cattle as the intermediate host and dog as the definitive host, respectively. Human affectation in most situations is accidental and asymptomatic. As a result, the slowly enlarging cysts in various organs remain unnoticed and neglected for years, eventually culminating in serious health consequences [1, 2].

Definitive treatment plan follows a combination of (1) imaging and immunologic studies for diagnosis, (2) a few courses of antihelminthic medications to sterilize the cyst, and/or (3) surgery or percutaneous drainage methods depending upon the response.

We hereby present a boy who had multiple giant hydatid cysts in the liver that were life-threatening and perilous in view of abnormally large dimensions and unusual locations.

## 2. Case Report

**Figure 1:**
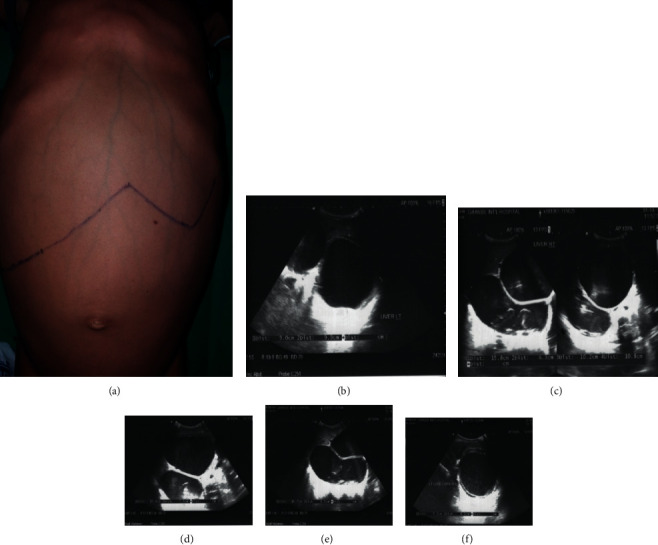
Massive hepatomegaly on clinical examination and ultrasonography showing 3 giant (≥ 10 cm) cysts involving both the liver lobes.

An 8-year-old boy from rural Nepal presented with progressive fullness and pain over the upper abdomen for a year without associated fever, jaundice, vomiting, or bowel complaints. There was no preceding history of trauma or weight loss. Physical examination revealed massive hepatomegaly, and ultrasonography showed 3 giant (≥10 cm) cysts involving both the liver lobes as shown in Figure 1.

Blood investigations were unremarkable, and echinococcal ELISA was negative.

**Figure 2:**
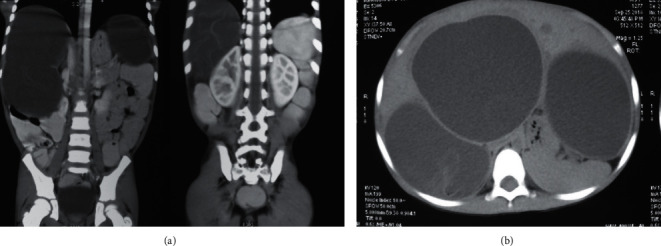
Abdominal CT scan showing multiple cysts with floating membranes in hepatic segments 2, 3, 6, and 7 with features favoring hydatid etiology.

Abdominal CT scan confirmed multiple cysts in hepatic segments 2, 3, 6, and 7 with features favoring echinococcal etiology as shown in Figure 2.

**Figure 3:**
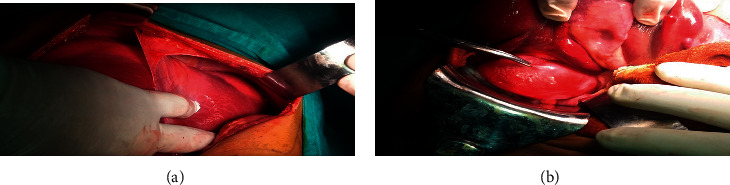
Intraoperative appearance of multiple liver hydatid cysts.

**Figure 4:**
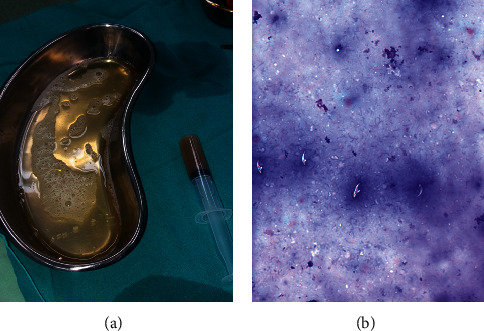
Contents of fluid aspirates from the hepatic hydatid cysts grossly and under microscopy showing free hooklets.

Following 3 weeks of oral albendazole therapy (10 mg/kg/day in 2 divided doses), he underwent laparotomy with cyst aspiration as shown in Figure 3. Aspiration revealed clear fluid from the 2 subphrenic cysts and bile-stained aspirates from the cyst over the under surface of the liver, cytology of which showed free hooklets as shown in Figure 4.

**Figure 5:**
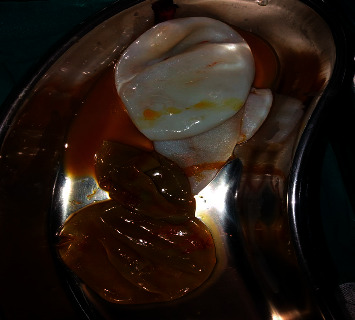
Non-bile-stained membranes within the subphrenic cysts and bile-stained ones in the cyst over the under surface of the liver.

Following this, instillation of the scolicidal agent (hypertonic saline) was done for 20 minutes, followed by reaspiration. The cysts were then opened, membranes evacuated as shown in Figure 5, and a careful search for a cyst-biliary communication was done, but not found.

Following this, partial pericystectomy with capitonnage and omentoplasty was done for cavity obliteration.

**Figure 6:**
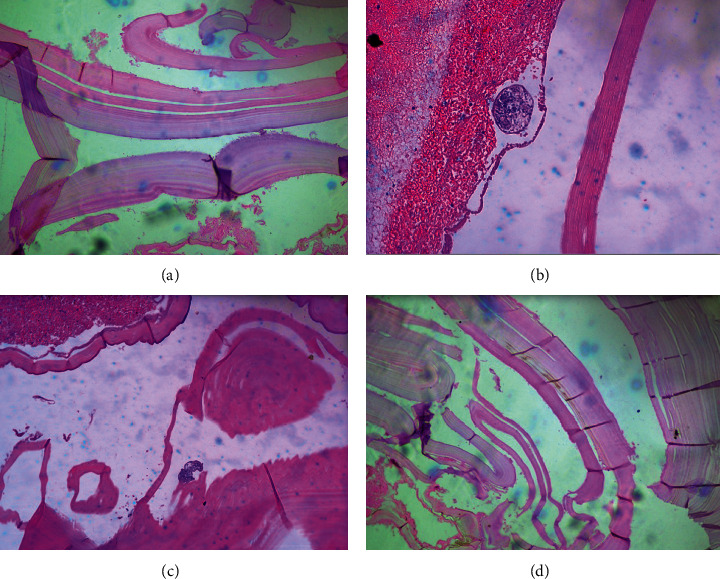
Histopathological appearance of the membranes showing laminated cuticular layer with protoscolex of *Echinococcus granulosus*.

Histopathology of the membranes showed laminated cuticular layer with protoscolex of *Echinococcus granulosus* as shown in Figure 6.

He had an uneventful postoperative recovery. The subhepatic and left subphrenic drains were removed on day 3 and day 4, respectively, and he was discharged on day 5.

At 2-week follow-up, his symptoms had improved, and he was advised to continue 2 further cycles of oral albendazole.

At follow-up, a year later, he remained symptom-free.

## 3. Discussion

Pediatric hepatic cysts are uncommon, of which the simple and solitary ones are mostly managed expectantly. However, multiple cysts require consideration of a broad range of pathologies. Amongst these, cystic echinococcosis is fairly common in the endemic areas of the developing world.

Although this can simultaneously involve multiple organs, in children the usual tendency is to affect the lungs as opposed to adults, wherein the liver is commonly involved [3–5].

The infection usually follows an indolent dormant course until reaching large sizes responsible for pressure symptoms [6]. The large cyst dimensions pose a significant potential for complications [7]. When the cyst attains a dimension greater than 5 cm, it is usually considered a giant hydatid cyst [8]. Though some cysts may respond to conservative treatment, the larger ones usually require surgical ablation [9].

The common hazards associated with large hepatic hydatid cysts are (1) traumatic or spontaneous rupture into the peritoneum leading to dissemination, recurrence, and secondary echinococcosis, (2) spontaneous intrabiliary rupture leading to cholangitis and sclerosis oddities, (3) trans diaphragmatic rupture into the pleural cavity, (4) erosion into the pericardial space causing effusion and tamponade, and (5) allergic reaction of varying severity due to fluid leak [6, 10, 11]. Similarly, intraoperative challenges include (1) cyst content spillage and later recurrence, (2) cyst-biliary communications posing technical difficulty in ligation, and (3) anaphylactic reactions that may be fatal.

While various modalities of management are described and also widely practiced, no definite guidelines exist concerning the age, cyst dimensions, and locations.

Laparoscopic techniques are most applicable to (1) superficial smaller cysts (<6 cm) over the anterior surface of the liver, (2) cysts without biliary communication, and (3) cysts ≤ 3 in number and are associated with risks of fluid spillage and subsequent recurrence or even intraoperative anaphylaxis [12].

Similarly, minimally invasive methods have also been described like PAIR (puncture, aspiration, injection, and reaspiration) that are mostly reserved for cysts that are puncture accessible, postsurgical relapse, unresponsive to drugs, or for those with surgical contraindications. However, the risk of secondary hydatidosis remains a concern requiring careful postoperative serological and image-based monitoring for these groups of patients [12].

With our patient, we opted for an open technique over the laparoscopic approach considering 2 specific problems: (1) subdiaphragmatic location of the cysts posing difficulty in laparoscopic access and (2) risk of transdiaphragmatic rupture into the lungs during manipulation due to cyst size that could result in sudden breathlessness and anaphylaxis. To deal with cysts of such giant dimensions and at unusual locations or with multiple lesions, in children, we prefer the open approach which also seems to be the method of choice in general [13].

Moreover, open approach provides additional advantage of cyst cavity obliteration using various techniques like omentoplasty or cyst capitonnage as was possible with our patient [14].

## 4. Conclusions

When dealing with multiple liver cysts in children, consideration for echinococcal etiology becomes important in endemic areas. Larger cysts require surgical ablation in general combined with antihelmithics. The preferred surgical approach depends upon the cyst dimension, location, and anticipated intraoperative events or complications.

## Data Availability

Data used to support the findings of this study are available from the corresponding author upon request.
